# Pre and Post-copulatory Selection Favor Similar Genital Phenotypes in the Male Broad Horned Beetle

**DOI:** 10.1093/icb/icw079

**Published:** 2016-07-01

**Authors:** Clarissa M. House, M. D. Sharma, Kensuke Okada, David J. Hosken

**Affiliations:** *Centre for Ecology and Conservation, College of Life and Environmental Sciences, University of Exeter, Penryn Campus, Cornwall, TR10 9EZ, UK; ^†^Laboratory of Evolutionary Ecology, Graduate School of Environmental Science, Okayama University, Tsushima-naka 1-1-1, Okayama, Japan

## Abstract

Sexual selection can operate before and after copulation and the same or different trait(s) can be targeted during these episodes of selection. The direction and form of sexual selection imposed on characters prior to mating has been relatively well described, but the same is not true after copulation. In general, when male–male competition and female choice favor the same traits then there is the expectation of reinforcing selection on male sexual traits that improve competitiveness before and after copulation. However, when male–male competition overrides pre-copulatory choice then the opposite could be true. With respect to studies of selection on genitalia there is good evidence that male genital morphology influences mating and fertilization success. However, whether genital morphology affects reproductive success in more than one context (i.e., mating versus fertilization success) is largely unknown. Here we use multivariate analysis to estimate linear and nonlinear selection on male body size and genital morphology in the flour beetle *Gnatocerus cornutus*, simulated in a non-competitive (i.e., monogamous) setting. This analysis estimates the form of selection on multiple traits and typically, linear (directional) selection is easiest to detect, while nonlinear selection is more complex and can be stabilizing, disruptive, or correlational. We find that mating generates stabilizing selection on male body size and genitalia, and fertilization causes a blend of directional and stabilizing selection. Differences in the form of selection across these bouts of selection result from a significant alteration of nonlinear selection on body size and a marginally significant difference in nonlinear selection on a component of genital shape. This suggests that both bouts of selection favor similar genital phenotypes, whereas the strong stabilizing selection imposed on male body size during mate acquisition is weak during fertilization.

## Introduction

Male primary and secondary sexual traits can be subject to sexual selection both before and after mating (Andersson 1984). The direction and form of pre-copulatory selection on males has been described for a number of taxonomic groups (reviewed in Hunt et al. 2009). Whereas the direction and form of selection on males during pre- and post-copulatory selection and how they interact has largely been neglected (but see Danielsson 2001; Evans et al. 2003; Head et al. 2006; Devigili et al. 2015). These bouts of sexual selection largely determine male fitness (Pischedda and Rice 2012; Pélissié et al. 2014; Mehlis et al. 2015) and estimating selection in each context is therefore important for our understanding of how episodes of selection reinforce or oppose one another and whether they favor the same or different phenotypes (Danielsson 2001; Hunt et al. 2009; Devigili et al. 2015).

When different episodes of selection target different traits, the availability of resources to allocate to pre- versus post-copulatory traits (Parker et al. 2013) or the genetic covariance between traits (Moore et al. 2004; Brown et al. 2009; Gay et al. 2011; Wagner et al. 2012) may limit the responses to selection. Sexual selection on a single trait can be complicated also, if for example, the direction or form of selection is reversed during pre- and post-copulatory events. Evidence of opposing pre- and post-copulatory selection has been found in the water strider *Gerris lacustris*. Large males have higher mating success but this is opposed by post-copulatory selection as small males’ secure higher fertilization gains (Danielsson 2001). In contrast, reinforcing selection for male body size has been found in the cricket *Acheta domesticus* (Head et al. 2006) and male body coloration in the guppy *Poecilia reticulata* (Evans et al. [2003]; although more recent evidence has identified further targets of selection see Devigili et al. [2015]). At present, too few studies are available to conclude that selection on single male traits that operate in more than one context is generally reinforcing or opposing.

Male genitalia are recognized as being among the most morphologically diverse and rapidly evolving structures in the animal kingdom despite their apparently simple function—delivering sperm (Eberhard 1985; 2010; Hosken and Stockley 2004). In arthropods, the male genitalia are multifaceted, with structures that specialize in clasping (secondary intromittent and secondary nonintromitent genitalia) the female to ensure secure genital coupling (Frazee and Masly 2015) and sperm transfer (primary intromittent genitalia). Eberhard (1985) proposed that sexual selection was responsible for this complexity and because differences evolve rapidly in closely related taxa, directional selection on genitals was thought to be particularly pervasive (Eberhard 1985; Arnqvist 1998). We can now say that Eberhard (1985) was right. Many single species studies show that variation in genital morphology influences reproductive success (Hosken and Stockley 2004; Simmons 2014) although the form of sexual selection on genitalia is often more complex than originally thought (Tadler 1999; Simmons et al. 2009; Wojcieszek and Simmons 2011; Dougherty and Shuker 2016). However, despite these advances, the direction and form of sexual selection imposed on the genital structures during pre- and post-copulatory events has received limited attention with the exception of studies in a beetle and bug (Simmons et al. 2009; Tadler 1999; Dougherty and Shuker 2016). In *Onthophagus taurus* pre- and post-copulatory sexual selection target different structures and forms of selection. Similarly, the comparatively simple intromittent organ of the seed bug is subjected to contrasting selection during pre- and post-copulatory sexual selection (Dougherty and Shuker 2016).

Male–male competition and female mate choice has been well studied in the horned beetle *Gnatocerus cornutus*. Males fight and fighting behavior is phenotypically and genetically correlated with male morphology (Okada and Miyatake 2009). Large males with large mandibles are competitively superior and monopolize access to females in competitive mating situations (Okada and Miyatake 2009; Harano et al. 2010; Yamane et al. 2010). However, during pre-copulatory sexual selection females prefer mating partners that court most vigorously rather than large competitive males (Okada et al. 2014). Furthermore, the dominant form of pre-copulatory sexual selection on male traits that have been measured (i.e., male body size, mandible morphology, and cuticle hydrocarbons) is stabilizing, such that intermediate male phenotypes secure more matings (Okada et al. 2014; Lane et al. forthcoming 2016; C. M. House, unpublished data). Therefore, during male–male competition and female mate choice it seems that selection on male traits is largely opposing. However we have a limited knowledge of selection on any other male traits that may influence the likelihood of successful copulation and how genital form could influence mating and fertilization success. Male genitalia are especially likely targets of post- and possibly pre-copulatory sexual selection (Hosken and Stockley 2004; Eberhard 2010). Currently we know that the male aedeagus is relatively insensitive to variation to nutrition compared with body and mandible size (House et al. 2015). As a consequence, good environments that increase male body and mandible size should have a limited influence on genital size. The relative canalization of genitalia seems to imply that sexual selection on genitals could be weak—there is not much variation on which selection could act—but this remains to be established.

Here we examined whether variation in male body size and the morphology of the intromittent organ of *G. cornutus* influenced mating and fertilization success during non-competitive matings (i.e., a monogamous setting) using standard multivariate selection analysis. While this is a simplification of selection on males during mating, it does reflect situations where larger males monopolize females by excluding rival males (Okada et al. 2014). We then compared the direction and form of selection during these different contexts to determine whether body size and the same genital characters were favored by pre and post-copulatory processes. It is often difficult to distinguish between male and female pre and post-copulatory processes as they often occur simultaneously (Birkhead 1998; Simmons 2001). Therefore, we eliminate male–male competition entirely, to explore whether female choice prior to copulation and male–female interactions during and after copulation favor the same male characters. This provides a useful baseline reference to investigate how male–male competition changes the patterns of selection on male characters in future studies.

## Methods

### Stock populations and rearing

Beetles were derived from the Japanese National Food Research Institute where they were established in 1957. In our laboratory, populations have been reared on whole meal flour (Doves Farms Foods Ltd) that is enriched with 5% yeast (ACROS organics), at 27 °C and 60% relative humidity under a 14:10 h light:dark cycle (see Okada et al. [2006] for details). Mixed sex populations consisted of 50 males and 50 female in each pot (*n* = 6; Thermoscientific Nalgene 500 mL, 120 mm OD) and at every generation larvae are randomly selected to form the parents of the subsequent generation (see House et al. [2015] for details).

To obtain adults for the present study, 144 final instar larvae were collected and individually placed in a single cell of a 24 well plate. Pupae were checked daily for eclosion, separated by sex and placed in single sex, 24 well plates to prevent interactions between individuals. All beetles were provided with approximately 1 g of whole meal flour enriched with 5% yeast and virgin males and females that were 11–15 days of age were used in the experiments described below.

### Experimental mating trials

#### Mating success

Virgin females were randomly selected and placed alone in a single cell of a mating arena (1 cm × 1 cm × 1 cm) lined with paper. After 10 min, a virgin male was added and the pair were observed for 20 min or until copulation occurred (*n* = 245), after which the male was removed. Of the males that did not mate, approximately 50% were observed to court (i.e., mount the female and drum her back rhythmically using his tarsi) (*n* = 255).

#### Fertilization success

Mating trials were conducted as above, using a new set of beetles that had the same range of phenotypes and were derived from the same stock population. Virgin females were randomly selected and placed in a single cell of a mating arena followed by a virgin male. Pairs were observed for 50 min or until a single copulation occurred. Females that mated were given the opportunity to oviposite in breeding pots (sized 67 × 34 mm) that contained 30 mL of wholemeal flour (Doves Farms Foods Ltd) enriched with 5% yeast (ACROS organics). On the 14th day, the females were removed and frozen (*n* = 508) and breeding pots were incubated. After 40 days, the number of offspring produced were counted.

The use of “no choice” mating assays ensured that male–male aggression which influences pre-copulatory selection on males (Okada and Miyatake 2009; Harano et al. 2010; Yamane et al. 2010) did not circumvent female choice and this is a standard method of assessing sexual selection (Koref-Santibanez 2001; Gowaty et al. 2002; Yenisetti and Hedge 2003; Shackleton et al. 2005). Therefore, by using this protocol, selection on the aedeagus could only be attributed to female choice/male attractiveness and/or mechanical constraints. However, it isn’t necessarily the case that selection on the aedeagus is equivalent during each episode of selection (i.e., mating success versus fertilization success) as mating does not mean fertilization (Eberhard et al. 2011). Indeed, we found that approximately one-fifth of our mated females produce no offspring and the remainder produce a range (i.e., 1–80).

### Measurement of morphological traits

After killing by freezing, experimental male beetles were placed on a slide, oriented consistently and digital images of the thorax were captured using a Leica M125 microscope with a mounted digital camera (Leica DFC 295) that was linked to a PC. Next, the aedeagus was removed from the abdominal cavity, placed on a glass slide so that the anterior tip of the aedeagus was laterally oriented to the left and then the whole structure was mounted in a droplet of Hoyer’s solution.

**Fig. 1:**
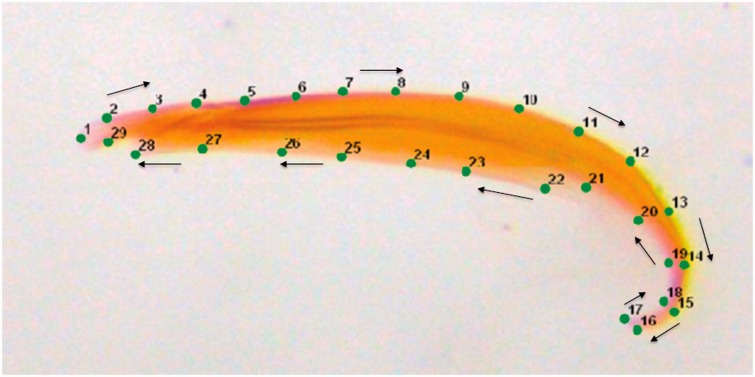
Geometric morphometrics (GMs) was used to capture variation in the size and shape of the aedeagus. To facilitate GM analysis, three landmarks (1, 16, and 17) and 26 semi-landmarks were placed along the outline of the aedeagus. The arrows indicate the order in which the landmarks were placed along the outline of the genitalia.

The width of the pronotum was measured as an index of body size (see Okada and Miyatake 2009) using Image J (version 1.48). Variation in the size and shape of the male genitalia was quantified using a geometric morphometric approach. First, three points that could be located precisely across all specimens were defined as fixed landmarks (type-two landmarks) and another 26 points were defined as semilandmarks as they slide along the curved outline of the aedeagus (Fig. 1). The fixed and semilandmarks were manually applied to all images in the same sequence (i.e., 1–29) in the program TPSDig 2.14 and the semilandmarks were identified by use of a “slider file” in the program TPSUTIL 1.46 (Rohlf 2009). Second, the landmark data were extracted in the program tpsRELW 1.46 (Rohlf 2008) and normalized for position, orientation, and scale (i.e., Procrustes superimposition) to eliminate non-shape variation (Zelditch et al. 2012). Next, centroid size (i.e., the square root of the sum of squared distances between landmarks from the central point of the specimen) and relative warp (RW) scores were calculated in tpsRELW 1.46. Finally, changes in the shape of the aedeagus were visualized as shape deformations of thin-plate spline plots in tpsRELW 1.46. The analysis was conducted on the complete data set (i.e., mating success and insemination success data combined), so that centroid size and the RWs were in the same geometric space to allow direction comparison of the direction and form of selection in two contexts. The 29 landmarks that defined the outline of the aedeagus yielded 54 RWs. Each RW explained diminishing amounts of variation in shape, so we only interpret RWs 1, 2, and 3 as cumulatively, they explain greater than 80% of the variance in shape (Gutierrez et al. 2011). It is possible that we have overlooked important variation in genital shape by limiting our analysis to that which is described by RW1, 2, and 3. However, the amount of variation that is described by subsequent RWs is small and difficult to describe as the shape changes are subtle. Furthermore, the interpretation of the strength and form of selection on traits can be troublesome as the number of traits increase and therefore the number of nonlinear terms increase (Hunt et al. 2009). Additionally, from a biological perspective, genital differences across taxa and populations are usually not subtle, in fact they are used to describe taxonomic differences when general morphology is identical. So using RWs that capture most of the variation seems the best approach from that perspective also.

We measured the repeatability of male body size and the repeatability of digitization of two images of the same male aedeagus using the R code provided in Wolak et al. (2012). The repeatability of body size and genital size and shape was high (Pronotum width = 0.989, 95% CIs = 0.985, 0.991; Genital size = 0.989, 95% CIs = 0.980, 0.998; RW1 = 0.936, 95% CIs = 0.887, 0.985; RW2 = 0.885, 95% CIs = 0.799, 0.971; RW3 = 0.795, CIs = 0.649, 0.941).

### Statistical analysis

#### Multivariate selection analysis

We used a standard multivariate selection analysis to estimate linear and nonlinear sexual selection on male body size and genitalia size (CS) and shape (RW1, RW2, and RW3) when virgin females were courted and/or mated or inseminated during a non-competitive mating (Lande and Arnold 1983). Mating success was assigned a binary fitness score of 0 if the male courted only (*n* = 255) and a score of 1 if the male courted and mated (*n* = 245). Fertilization success was assigned a continuous fitness score that was the total number of offspring that a male sired as we reasoned that after single copulations variation in offspring number would be influenced by successful ejaculate transfer. We note that if variation in aedeagus form does not influence this or is only one element in the pathway between insemination to offspring production, we would be unlikely to detect selection on the aedeagus (i.e., our assumption is conservative). For each bout of selection (i.e., mating success (_ms_), versus fertilization success (_fs_)), we transformed fitness scores to a mean of one (i.e., relative fitness) and all male phenotypic traits to zero means and unit variances (i.e., standardized traits) following the recommendations of Lande and Arnold (1983). We then used multivariate linear and polynomial regression models in each selective context to estimate linear and nonlinear (i.e., quadratic and correlational) selection gradients for male size and genital size and shape during mating (*β*_ms_ and γ_ms_) and insemination (*β*_fs_ and γ_fs_) (Lande and Arnold 1983, see Hunt et al. 2009 and Lewis et al. 2011 for details). We doubled the quadratic selection gradients as stabilizing or disruptive selection is underestimated by a factor of 0.5 (Stinchcombe et al. 2008).

To assess the significance of our linear and nonlinear selection gradients for each dataset we used a resampling procedure (Mitchell-Olds and Shaw 1987). Fitness scores were randomly shuffled across individuals in the dataset so that there was no relationship between our traits and fitness (Mitchell-Olds and Shaw 1987). Then we used Monte Carlo simulation to determine the proportion of times (out of 9999 permutations) that the gradient pseudo-estimate was equal to or less than the original estimated gradient and we used this to calculate a two-tailed probability value for each selection gradient in the model (Manly 1997). Separate randomization analyses were undertaken for the linear and polynomial regression models (see Hunt et al. [2009] and Lewis et al. [2011] for details).

Individual γ-coefficients are likely to underestimate the strength of nonlinear selection overall (Phillips and Arnold 1989; Blows and Brooks 2003). To determine the extent of nonlinear sexual selection we conducted a canonical analysis to locate the major axes of multivariate nonlinear selection during mating and fertilization (Phillips and Arnold 1989; Reynolds et al. 2010). This analysis generates an M-matrix, which contains eigenvalues and their corresponding eigenvectors (*m_i_*) with the overall strength of linear selection given by theta (*θ_i_*) and nonlinear selection given by the eigenvalue (λ_*i*_). The magnitude and sign of the *m* scores describe how the original traits covary with each other and their relative contribution to an eigenvalue. To test the significance of the eigenvalues we followed a permutation procedure outlined in Reynolds et al. (2010). This procedure does not test the significance of linear selection across the eigenvectors, so instead we used a “double regression” to test the strength of linear selection across these eigenvectors (Bisgaard and Ankenman 1996).

Thin-plate splines (Green and Silverman 1994) were used to visualize the major axes of selection for γ_ms_ and γ_fs_ against the relative fitness of males (see Blows and Brooks 2003; Blows et al. 2003; Hall et al. 2008; Stinchcombe et al. 2008 for detailed examples of these techniques). The Tps function in the fields package of R (version 2.13.0; available via http://www.r-project.org) was used to fit the splines using a value of the smoothing parameter that minimized the generalized cross-validation score (Green and Silverman 1994) and presented the perspective view of the surfaces.

Finally, we used a sequential model building approach (partial *F*-test) to compare the strength and form of sexual selection on male traits during mating and fertilization (Draper and John 1988; see Chenoweth and Blows [2005] for a detailed description of this procedure).

## Results

**Fig. 2:**
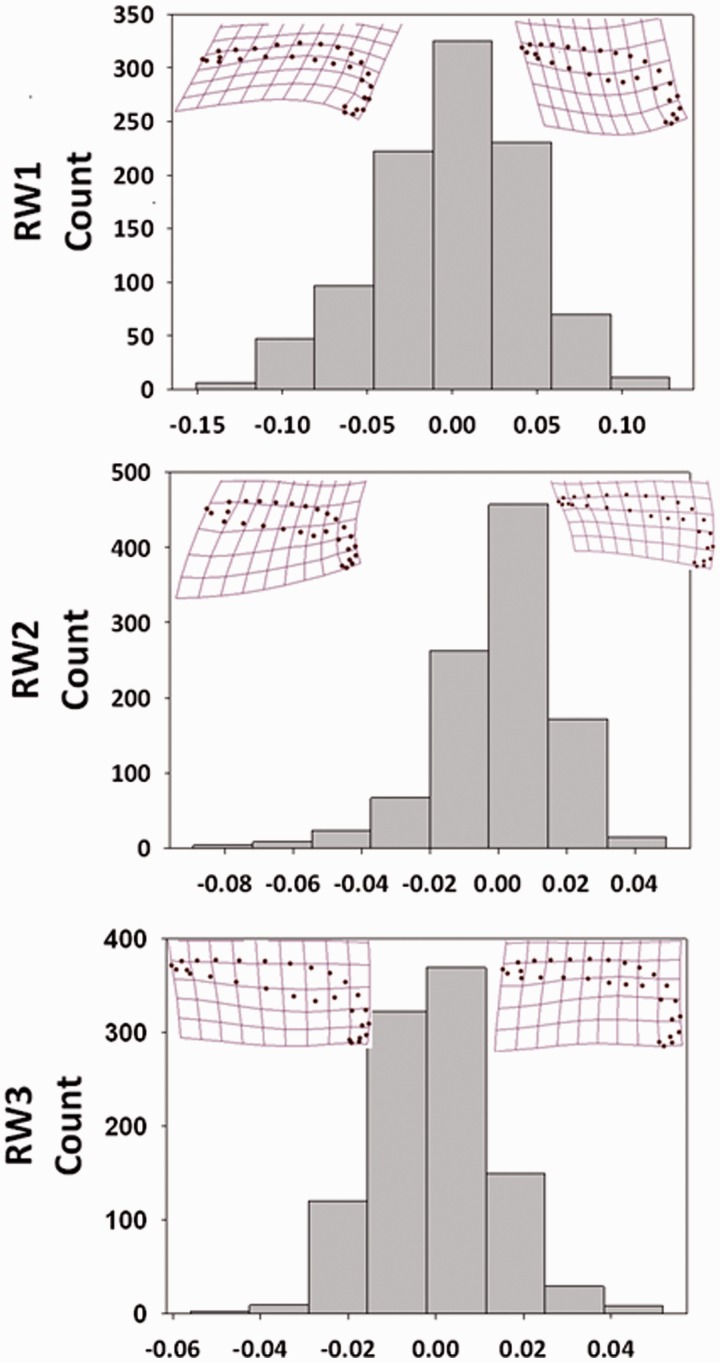
Frequency distribution of the three relative warp (RW) scores characterizing the variation in male intromittent organ shape. Thin-plate spline visualizations that characterize a positive and negative RW score are inset for each RW. 65.3% of the shape variation was explained by RW1, 11.4% by RW2, and 6.7% by RW3.

Shape analysis of 1008 males across our two datasets yielded three RWs that explained 83.34% of the variation in the intromittent organ. RW1 explained 65.27% of the total variation in genital form with positive values corresponding with a thicker midsection and negative values corresponding with thin midsection (Fig. 2). RW2 explained another 11.37% of the variation with positive values corresponding with a narrow anterior tip and elongated midsection and negative values corresponding with a wide anterior tip and short midsection (Fig. 2). RW3 explained a final 6.70% of the variation with positive values corresponding with a longer posterior section that is the point of attachment to the male internal structures and negative values corresponding with a shorter posterior section (Fig. 2).

Standardized linear, quadratic, and correlational selection gradients for body size, intromittent organ size and shape during mating and fertilization success are presented in Table 1. For mating there is significant negative directional selection on RW1 (thin midsection of the intromittent organ) and positive correlational selection on genital size and RW1 and RW1 and RW3. For fertilization there was significant negative directional selection on genital size and RW3 (shorter posterior section) and positive directional selection on RW2 (narrow anterior tip and elongated midsection). There was also significant stabilizing selection on genital size and RW3 and positive correlational selection on body size and RW3.

**Table 1:** The vector of standardized linear selection gradients (*β*) and the matrix of standardized quadratic and correlational gradients (*γ*) for body size and genital size and shape in male *G. cornutus* during (A) mating success and (B) fertilization success

		*Γ*				
	*β*	PW	CS	RW1	RW2	RW3
A. Mating success						
PW	−0.014	−0.146				
CS	−0.017	−0.016	−0.214			
RW1	−0.117*	−0.058	0.201*	−0.196		
RW2	0.005	0.048	0.077	0.100	−0.146	
RW3	−0.052	0.061	−0.180	0.159*	0.012	−0.142
B. Fertilization success						
PW	0.081	−0.086				
CS	−0.195**	0.178	−0.340*			
RW1	0.020	−1.533	0.139	−0.048		
RW2	0.074*	0.003	−0.028	0.072	0.078	
RW3	−0.104*	0.177**	−0.131	0.064	0.014	−0.190*

*Note*: Randomization test:

*P < 0.05,

**P < 0.01,

***P < 0.001.

**Fig. 3:**
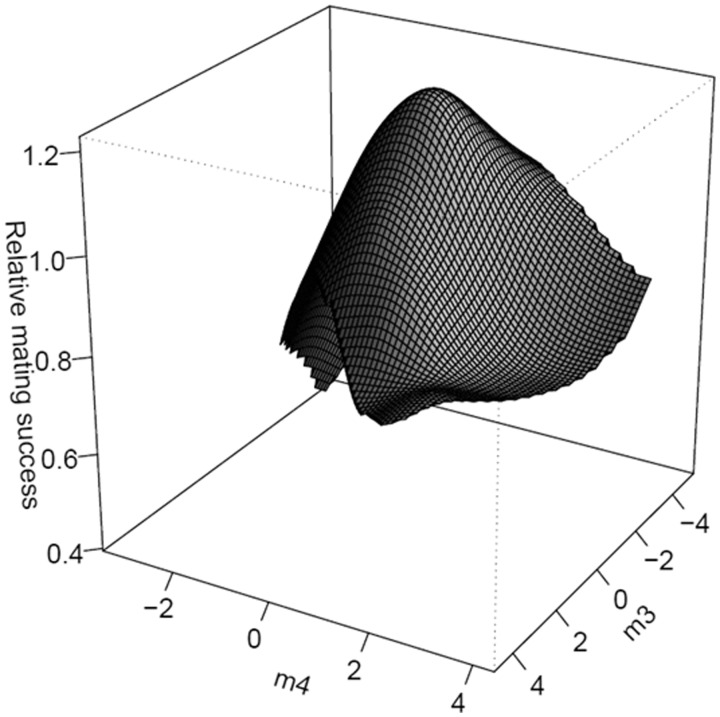
Thin-plate spline visualizations (perspective view) of the two major axes of nonlinear selection (**m_3_** and **m_4_**) on the fitness surface during mating success.

**Fig. 4:**
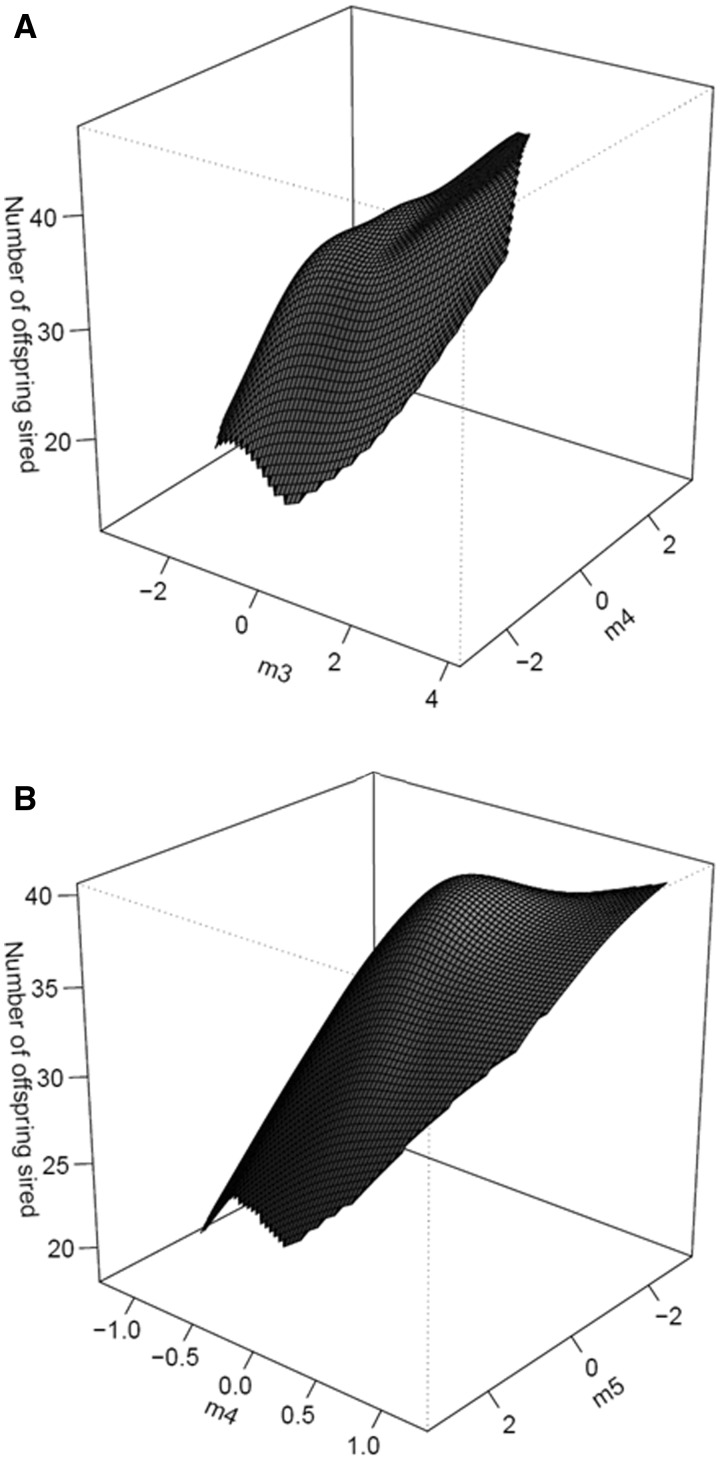
Thin-plate spline visualizations (perspective view) of the two major axes of linear and nonlinear selection for **(A) m_3_** and **m_4_** and **(B) m_4_** and **m_5_** during fertilization success.

Our selection analysis to identify the major dimensions of nonlinear selection on body size, genital size and shape, during mating and fertilization is presented in Table 2. During attempted mating, directional selection was non-significant for all eigenvalues. Nonlinear selection for eigenvalues was a mixture of positive (**m_1_** and **m_2_**) and negative (**m_3_**, **m_4_**, and **m_5_**) values but significant nonlinear selection was only detected on **m_4_** and **m_5_** indicating that selection on the fitness surface is stabilizing (Fig. 3). During fertilization, directional selection was a mixture of positive (**m_1_**, **m_3_**, **m_4_**, and **m_5_**) and negative (**m_2_**) values but significant directional selection was only detected on **m_3_** and **m_5_**. Directional selection on **m_3_** was primarily loaded on RW1 and RW2 whereas on **m_5_**, selection was strongest and primarily loaded on CS and RW3. Nonlinear selection for eigenvalues was a mixture of positive (**m_1_** and **m_2_**) and negative (**m_3_**, **m_4_**, and **m_5_**) values but significant nonlinear selection was only detected on **m_4_** indicating a mixture of directional and stabilizing selection on the fitness surfaces for the statistically significant eigenvectors, **m_3_**, **m_4_**, and **m_5_** (Fig. 4A, B).

**Table 2:** The **M** matrix of eigenvectors from the canonical analysis of ***γ*** for (A) mating success and (B) fertilization success in male *G. cornutus*

	M											
	PW		CS		RW1		RW2		RW3		*θ_i_*	*λ_i_*
A. Mating success												
**m_1_**	−0.120		0.604		0.627		0.474		−0.050		−0.030	0.072
**m_2_**	0.291		−0.335		0.307		0.180		0.822		−0.110	0.014
**m_3_**	**0.799**		0.079		−0.290		**0.459**		−0.244		0.047	−0.117*
**m_4_**	**0.492**		**0.389**		0.273		−**0.728**		0.042		−0.020	−0.257**
**m_5_**	−0.140		0.604		−0.595		0.035		0.510		0.095	−0.557
B. Fertilization success												
**m_1_**	0.022		0.066		0.473		0.869		0.123		0.050	0.118
**m_2_**	0.847		0.224		−0.010		−0.100		0.472		−0.032	0.061
**m_3_**	0.080		−0.390		−**0.794**		**0.443**		0.118		0.087*	−0.023
**m_4_**	0.283		**0.542**		−0.241		0.186		−**0.730**		0.002	−0.144*
**m_5_**	0.443		−**0.707**		0.294		−0.052		−**0.463**		0.224**	−0.600

*Notes*: The linear (*θ_i_*) and quadratic (*λ_i_*) gradients along each eigenvector are given in the last two columns. The quadratic selection gradient (*λ_i_*) of each eigenvector (m_i_) is equivalent to the eigenvalue. Randomization test:

*P < 0.05,

**P < 0.01,

***P < 0.001.

To test for possible differences in selection on male body size and genital morphology during mating versus fertilization we compared the strength and form of directional, quadratic (i.e., negative quadratic selection is stabilizing selection and positive quadratic is disruptive selection), and correlational selection across selective bouts. The strength of directional (*F*_5,996 _ = 1.171, *P* = 0.321) and correlational selection (*F*_10,966 _ = 0.940, *P* = 0.495) did not differ significantly between these bouts of selection, whereas quadratic sexual selection significantly differed between the selection episodes (*F*_5,986 _= 3.156, *P* = 0.008). In the case of quadratic selection, this was generated by a significant difference in selection on body size (*F*_1,986 _ = 10.605, *P* = 0.001), a marginally significant difference in quadratic selection on RW2 (*F*_1,986 _ = 3.699, *P* = 0.055), but not by differences in CS (*F*_1,986_ = 2.162, *P* = 0.142), RW1 (*F*_1,986 _ = 0.943, *P* = 0.332), or RW3 (*F*_1,986_ = 1.483, *P* = 0.224).

## Discussion

This study provides insights into the complexity of sexual selection on males before and after mating. We estimate selection under conditions that approximate a female’s first mating in the absence of competition and there was precopulatory selection on body size, and selection on the intromittent organ morphology during both pre- and post-copulatory sexual selection. Sexual selection was stabilizing along the major axes of multivariate selection during the mating phase (pre-copulation) and was largely due to the success of males with intermediate body size. In contrast, multivariate selection on the penis was similar during each selective episode (i.e., a mix of directional and stabilizing selection) and favored largely the same genital phenotype. We do not know how much of the variation in male reproductive success is due to pre- or post-copulatory processes (but see Hosken and House 2011; Pischedda and Rice 2012), but it is evident that both episodes of sexual selection can potentially contribute to the evolution of the male phenotype. Furthermore, even under the rather simple paradigm explored here, our findings suggest that selection favors intermediate sized males but because the genitalia of *G. cornutus* are relatively canalized (House et al. 2015), extreme male phenotypes are also likely to have genitalia that are effective at gaining fertilizations.

Pre-copulatory selection imposed stabilizing selection on **m_3_** (loaded strongly by body size and RW2) and **m_4_** (loaded strongly by body size and RW2) so that average male body size and an intermediate shaped anterior tip and midsection of the intromittent organ were favored. This could be attributed to either active female preference or mechanical constraint (passive female choice: Wiley and Poston 1996). Female mate choice for average sized males during mating may reflect a strategy to avoid larger, competitive male phenotypes that sire low fitness daughters due to intralocus sexual conflict (Harano et al. 2010; Katsuki et al. 2012; Okada et al. 2014). The daughters of competitive males have decreased fitness as the genes that make males good fighters also masculinize females and these females are less fecund (Harano et al. 2010). In contrast, the mechanisms of sexual selection that impose selection on the male intromittent organ during mating success are unknown. In the seed beetle *Lygaeus equestris* the form of pre-copulatory selection on components of the male genitalia was stabilizing as we find here (Dougherty and Shuker 2016). However, the authors conclude that this pattern is due to selection on a correlated, unmeasured trait as the genital capsule is stored internally before mating (Dougherty and Shuker 2016). In this study, we cannot rule out this possibility, but during courtship the male intromittent organ is extended externally and interacts with the female when the male contacts the female genital opening—as is found in a dung beetle and waterstrider although selection on the aedeagus is directional in these species (Preziosi and Fairbairn 1996; Ferguson and Fairbairn 2000; Sih et al. 2002; Bertin and Fairbairn 2005; Simmons et al. 2009). The flour-beetle data are more consistent with Eberhard’s one-size-fits-all argument, which posits that intermediate genitalia of males are favored as they better fit the average female size in the population (Eberhard et al. 1998). Genital allometry tends to support this idea too, even in taxa with exaggerated genitals (e.g., Hosken et al. 2005; Higgins et al. 2009) and the stabilizing selection we find here would explain this negative allometry. The weak responsiveness of the developing intromittent organ of *G. cornutus* to variation in nutrition results in relatively little phenotypic variation in genital size, a pattern that fits the one-size-fits-all arguments of Eberhard et al. (1998). However, this pattern is more difficult to reconcile with directional selection on the size of the genitalia that we find during fertilization success (see below). Unless, directional selection for small genitals is proportionally stronger in large males than smaller males (Dreyer and Shingleton 2011).

Post-copulatory selection along the vectors of strongest selection imposed stabilizing selection on **m_4_** (loaded strongly by genital size and RW3) and directional selection on **m_3_** (loaded strongly by RW1 and RW2) and **m_5_** (loaded strongly by genital size and RW3). Previously we have found that the morphology of the intromittent organ was only weakly related to body size (House et al. 2015) and it is therefore unsurprising that selection on body size is relatively weak during fertilization. Sexual selection acting on the genitalia favored a small aedeagus with a narrow anterior tip (+ ve RW2), thin midsection (−ve RW1) and shorter, curved posterior section (−ve RW3) at the fitness peak. Beyond the peak on the fitness landscape, selection was stabilizing and further reductions in size or shape of the posterior section were not favored. Interestingly, directional selection on individual components of the size and shape of the intromittent organ were significantly similar across selective bouts, and, although, quadratic selection differed, the contribution of a single genital component to this change in selection was small. Overall, this suggests that selection favors a similar intromittent organ phenotype during mating and fertilization although the individual gradients tended to be larger during fertilization success. It is possible that this pattern is confounded with other factors such as female fertility and genetic incompatibilities. However, because we randomly assigned females to males these effects should not be systematically biased in any particular direction with respect to genital size and shape. Our estimates could also be biased by selection acting on other male traits correlated to genitals (i.e., testis size and sperm number) that we did not measure. This is a general issue with all selection estimates and one that can never be solved unless we can find a way to measure the full phenotype in a biologically relevant way. Furthermore, whether this pattern holds when male–male competition and sperm competition is included in selection estimates remains to be established.

Correlational studies in a range of insects (reviewed in Simmons 2014) and a millipede (Wojcieszek and Simmons 2011) have shown that male genitalia are subject to post-copulatory sexual selection as Eberhard consistently argued would be the case (Eberhard 1985). The majority of studies have found evidence of directional selection acting on genital traits during non-competitive (Holwell et al. 2010) and competitive (Arnqvist and Danielsson 1999; Cordoba-Aguilar 1999, 2002; 2009; Danielsson and Askenmo 1999; Wenninger and Averill 2006; Van Lieshout 2011; Van Lieshout and Elgar 2011) fertilization success with the exception of studies in a seed bug (non-competitive mating; Tadler 1999; Dougherty and Shuker 2016), dung beetle (competitive mating; Simmons et al. 2009), and millipede (competitive mating; Wojcieszek and Simmons 2011) where stabilizing selection has been found to act. However, as is generally the case, most genital studies have only tested for directional selection. In *G. cornutus* we can only speculate why males with a smaller intromittent organ and certain genital shape combinations have higher mating and fertilization success in the absence of competition. Perhaps the intromittent organ anchors into the female last abdominal segment and stabilizes copula (i.e., holdfast device), stimulates the female internally (i.e., cryptic female choice), or enables males to thwart female control—these alternatives deserve further study.

Here we limit our study to the investigation of pre- and post-copulatory selection on genitalia and do this within a simple experimental regime that provide a “baseline” estimate of selection in the absence of male–male competition. However, we know that a number of other traits (i.e., mandible size and CHCs) are important for male sexual fitness. Theoretically, the allocation of resources to traits that are important prior to mating may limit allocation to traits that are important post mating (Parker et al. 2013). The findings of studies that have tested for the predicted negative covariance between pre- and postcopulatory traits are inconsistent. Tradeoffs are evident in some systems and not others and one explanation is that the strength of male monopolization of a female predicts the strength and direction of the covariance between sexual traits (Lupold et al. 2014). In taxonomic groups where males monopolize a female(s) the negative covariance between pre and postcopulatory sexual traits is stronger compared with taxonomic groups where female monopolization is less common (Lupold et al. 2014). In *G. cornutus*, competitive males can monopolize females and female *G. cornutus* are promiscuous (Okada et al. 2015) so it seems likely that selection on male traits will change when pre- and postcopulatory male–male competition is included and this is an area of research we wish to address next.

## Conclusions

### Consequences of interacting mechanisms of sexual selection

We know that sexual selection on male traits that is imposed by male–male competition and female mate choice can be reinforcing or opposing (Hunt et al. 2009). Females should prefer dominant males and the traits that confer dominance when males provide net benefits (Wong and Candolin 2005). Conversely, if mating with dominant males incurs a net cost, female mate choice can impose opposing selection on traits that are important for dominance—even though female mate choice may be overridden (Wong and Candolin 2005). In much the same way, it is possible that pre- and postcopulatory mechanisms of sexual selection can impose equally complex selection on male traits. Empirical evidence suggests that selection on male traits during pre- and postcopulatory sexual selection can be reinforcing, suggesting that male and female interests are aligned, but not always (Danielsson 2001; Evans et al. 2003; Head et al. 2006; Devigili et al. 2015). The important general point is that the form of selection that is imposed on male sexual traits following a single bout of selection may not be the same in another (Wong and Candolin 2005; Andersson and Simmons 2006; Hunt et al. 2009). Therefore, through the study of selection across different selective bouts we can begin to understand how sexual selection drives the evolution of male phenotypes.
